# Psychometric Properties of the Moral Disengagement through Technologies Questionnaire (MDTech-Q) in a Sample of Chilean University Students

**DOI:** 10.3390/healthcare11081097

**Published:** 2023-04-12

**Authors:** Karina Polanco-Levicán, Sonia Salvo-Garrido

**Affiliations:** 1 Programa de Doctorado en Ciencias Sociales, Universidad de La Frontera, Temuco 4780000, Chile; k.polanco01@ufromail.cl; 2 Departamento de Psicología, Universidad Católica de Temuco, Temuco 4780000, Chile; 3 Departamento de Matemática y Estadística, Universidad de La Frontera, Temuco 4780000, Chile

**Keywords:** moral disengagement, Internet, social networks, university students, measurement

## Abstract

In the virtual environment, hostile and aggressive comments that could negatively affect university students who often use different digital platforms are frequently observed, more than other age groups who have little or no supervision. In this sense, moral disengagement (MD) has been linked to different negative behaviors that manifest in physical interactions and which currently take place on the Internet, creating the need for instruments that specifically address MD online. The aim of this study is to adapt and validate the Moral Disengagement through Technologies Questionnaire (MDTech-Q) with Chilean university students. The sample comprised 527 university students (43.14% men, 56.86% women), with an average age of 22.09 years (SD (Standard Deviation) = 3.59) enrolled in 12 universities. First, a linguistic adaptation of the scale took place, and the surveys were applied considering ethical principles. Then, two confirmatory factor analyses (CFA) were performed, which considered four correlated factors, and provided satisfactory indices, agreeing with the original theoretical proposal, and demonstrating suitable reliability by internal consistency. In relation to the analyses of invariance according to sex and social media use, the MDTech-Q is stable up to scalar invariance. This study provides evidence of the psychometric quality of the MDTech-Q for its use on Chilean university students.

## 1. Introduction

Various aggressive behaviors are observed on the Internet that cause concern nationally and internationally, given that cyberspace has become one of the most common contexts for people to function [1,2]. In this sense, university students spend long periods of time on social networks. It is reported that 74.3% of the students in a sample in Algeria use social networks for more than three hours a day [3], while Hispanic college students report an average of 20 h a week [4], and 98.3% of a sample of Brazilian students have at least one social media account [5]. For university students, social networks and other digital platforms are frequently used to communicate, inquire, and interact [6,7], but the experiences can be positive and negative. On the one hand, they favor the formation of friendships, reduce feelings of loneliness, offer social support, and lift a person’s mood; on the other, there are hostile and hate-filled comments, self-denigrating comparisons and aggressions that are part of the risks that could emerge when using these communication tools [8,9,10,11]. Moral disengagement (MD) has been related to different aggressive behaviors in both cross-sectional and longitudinal studies; however, greater evidence is required on the role of MD through technology [12,13,14,15].

According to Bandura et al. [16], MD focuses on the psychological processes that enable the cognitive and affective reconstruction of the transgression. These socio-cognitive and self-regulation strategies allow people to manifest immoral behaviors while at the same time justifying their harmful behavior [16,17]. Moreover, these strategies are used to protect the self-esteem from the negative affect produced by executing actions in conflict with the person’s moral values [18]. It is important to mention that behavior in the initial stages of development is regulated by external social standards and sanctions; however, in the course of socialization, people progressively adopt moral standards about what they consider correct and incorrect that guides their behavior and, in the case of a misalignment, self-sanctions emerge [18]. In this process, behavior is self-monitored and evaluated according to the moral standards and characteristics of the situation. In addition, it is regulated by virtue of the anticipation of the positive and negative outcomes that a particular behavior entails; in this sense, it encourages the action to be in line with the moral values [18,19]. However, the self-regulation mechanisms must be activated and the moral self-sanctions can be disconnected from the behavior that people deploy; therefore, activation and selective disengagement make it possible for different behaviors linked to the same moral standards to appear [18].

Thus, Bandura et al. [16,18] proposes that the main points of the self-regulation process in which selective disengagement of internal control could occur, favoring the implementation of negative behavior, are linked to four rationalization techniques comprised of eight MD mechanisms, which are the following. First, the negative behavior could be reconstructed cognitively by transforming it into less of a wrong-doing to avoid the emergence of self-sanctions. The mechanisms that are part of this strategy are: moral justification, euphemistic labeling, and advantageous comparison. Second, the strategy concentrates on the responsibility of the action, minimizing the role of the person who performs the negative behavior. The mechanisms that are part of this strategy are: transference of responsibility and diffusion of responsibility. Third, the strategy focuses on the consequences of the behavior, where people can distort, minimize, or disregard the impact their actions have on others. This mechanism is called distortion of consequences. Fourth, the strategy concentrates on the victim; therefore, it is based on devaluing and laying responsibility for the detrimental behavior suffered. The mechanisms that reflect this strategy are: attribution of blame and dehumanizing the victim.

MD is linked to different negative behaviors such as online hate speech [20], racist behavior on news websites [21], hostile emotions [22], sexist memes online [23], cyberbullying [24,25,26,27], and increased cyberaggression and cybervictimization [28,29,30]. In addition, it has a moderating role between social network fatigue and online trolling [31]. Specifically, Lee & Jang [1] report that some of the predictors of cyberaggression in university students are anonymity, being male, a lower level of moral justification, a higher level of advantageous comparison, distortion of consequences, and attribution of blame. In the same vein, MD is a relevant risk factor for those students who have been cybervictimized since it contributes to the possibility of moving to the role of cyberaggressor [32]. In the case of the spectators in cyberbullying, the mechanisms of MD and moral justification are related to passive observation [33,34]. Therefore, MD favors the understanding of the cognitive mechanisms that promote the appearance of negative behaviors online such as bullying [14].

In this way, university students have more opportunities to use MD because they are on the Internet and have access to social networks without supervision or restrictions, and they could frequently avoid the sanctions that the negative behavior exerted physically entails [27,35,36]. In addition, emerging adults could activate this cognitive mechanism more quickly and easily given the greater development of cognitive abilities compared to previous stages of development [24]. Another relevant aspect is that studies have described MD as being linked to sex, with higher scores being shown in men [37,38,39]. In this sense, there is evidence of differences in favor of men over women in relation to MD and cyberbullying linked to sociocultural aspects like the way problems are addressed and solved [40,41,42,43,44]; however, results on the matter are contradictory [29,44,45,46,47].

However, Bandura [48] posits that virtual spaces favor disconnection from moral self-sanctions due to the difficulties in supervising and regulating people’s behavior on the Internet. It is noteworthy that interactions through electronic devices have characteristics that could favor a display of negative behaviors such as the scarcity and difficulties of recognizing and interpreting socio-emotional signals, anonymity, distance in time and space, among others [27,28,29,30,31,32,33,34,35,36,37,38,39,40,41,42,43,44,45,46,47,48,49,50,51,52,53,54,55]. Wang & Ngai [56] refer to anonymity and asynchronicity as being related to the perpetration of cyberbullying through MD. On the other hand, the limited access to socio-emotional signals and the rapid and easy dissemination of information could be linked to the mechanism of moral justification, euphemistic labeling, advantageous comparison, diffusion of responsibility, distortion of consequences, dehumanization, and attribution of blame. This could indicate that the Internet context could cause MD and, therefore, aggression [13,57].

Thus, to truly understand MD, it is relevant to understand moral behavior in cyberspace and its relation to negative and aggressive behaviors [57,58,59]. In this way, traditional MD could be connected to the rise in deviant behaviors in different contexts; however, online MD responds particularly to the specific features of the virtual environment [43]. According to Paciello et al. [43], traditional MD is associated with people with high externalization issues, whereas online MD is related to cyberbullying in people who do not present risks of manifesting these behaviors in face-to-face interactions; therefore, they prioritize the conditions of the virtual environment, helping people to become disengaged morally, finding that the traditional and online MD are correlated. It should be noted that in terms of the MD produced in interactions through technology, it is essential to analyze social and educational phenomena such as cyberbullying; therefore, it is important to use measures of MD that are appropriate to assess these cognitive mechanisms online [13,43,60].

The Moral Disengagement through Technologies Questionnaire (MDTech-Q) was developed by Marín-López et al. [13] and evaluates the different MD mechanisms people use while they interact online. This instrument is based on the proposal by Bandura et al. [16,18] and assesses four of the MD mechanisms raised by this author, which are: Moral Justification, Diffusion of Responsibility, Distortion of Consequences and Attribution of Blame. Each of these mechanisms is linked to four different strategies that deactivate moral self-censoring of the person’s behavior. First, the redefinition of the harmful behavior, second, the minimization of the role played and the diffusion of responsibility of the aggressive action to the group, third, distortion of the damage or impact that the behavior has had on others and, fourth, the resignification of the action by blaming the victim. It should be mentioned that, considering the prolonged periods on different digital platforms on the Internet, it is important to develop an appropriate measure to assess MD through technology, given the evidence that online MD is different from traditional MD [13,43].

In the same vein, it is noted that this scale has good psychometric properties. Specifically, it presents good validity indicators for the structure of four correlated factors in a population of Spanish adolescents (SB-χ^2^ (103) = 242.14 *p* < 0.001; RMSEA (root mean square error of approximation) = 0.04 (90%CI (confidence interval) = 0.03–0.04); CFI (comparative fit index) = 0.99; NNFI (non-normed fit index) = 0.99; NFI (normed fit index)= 0.99) in addition to adequate reliability (Justification through Technology: α = 0.91; Diffusion of Responsibility through Technology: α = 0.91; Distortion of Consequences through Technology: α = 0.91; Attribution of Blame through Technology: α = 0.86). It should be stressed that previous studies about the Traditional Moral Disengagement construct had proposed a unidimensional structure [16,61,62,63]; however, Pozzoli et al. [64], coincidently with the findings of the original study by Marín-López et al. [13,65], have demonstrated a model of four correlated factors because each of the dimensions represents a place in the self-regulation system in which the negative behavior could be disconnected from the inner moral control. It is relevant that there are no adaptations or validations of this scale in other populations; therefore, this is another element that lends importance to this study.

In terms of the application of this scale, Marín-López et al. [65] in a study with Spanish adolescents, describe MD through technology as being related positively to cyberbullying, noting that greater cybervictimization and cyberaggression are associated with higher scores of MD through technology, specifically cyberaggressors/victims show higher levels of MD through technology than the victims and uninvolved spectators. In the case of cyberaggressors, higher scores are noted in both cross-sectional and longitudinal studies. The mechanism of moral justification stands out as it considered a risk factor for the role of aggressor in cyberbullying, demonstrating that the scores remain high in this dimension a year after the first measurement [65].

The relevance of this adaptation and validation with Chilean university students is linked to the existing difference between traditional MD and the MD that occurs in the interactions on the Internet related to the characteristics of this context [13,43]; therefore, it offers an instrument designed to address the cognitive mechanisms of MD since aggressive and negative behaviors are frequently observed on the Internet. After this, it should be noted that the development, adaptation, and validation of a scale that evaluates the MD that arises in interaction through screens among Chileans has not been observed. Therefore, this study will offer evidence of the factor structure and psychometric properties of this instrument in Chilean university students considering the cultural differences with the Spanish population that would be reflected in the way opinions are expressed [66], and the way misuse of information on the Internet is addressed [67]. It must also be borne in mind that in Latin America there are divides in terms of poverty and access to Internet [68,69]. It is important to point out that Latin America has social, economic, educational, political, and cultural conditions that show a region oriented toward the acquisition rather than the generation of new technologies, which may have consequences in the timely approach to problems that occur on the Internet [70]. In the present context, where different digital platforms are often used, it is very important to aid in responsible Internet use to prevent different negative behaviors considering that 78.3% of a sample of Chilean university students use social networks daily [71]. To do this, it is essential to remember the mechanisms of MD in order to implement strategies that reduce the possibility that people will disconnect sanctions and moral values from the manifested behavior [1].

Based on this and on the theoretical and empirical relevance of the MD construct and its application to technology and on unidimensional [16,61,62,63] and correlated four-factor structures [13,64,65], two hypotheses are posited: the first establishes that the scores on the MDTech-Q will present a unidimensional factor structure or four correlated factors called: moral justification through technology, diffusion of responsibility through technology, distortion of consequences through technology, and attribution of blame through technology, and adequate levels of reliability for the Chilean context. The second hypothesis proposes that the scores on the MDTech-Q would stay steady up to the level of scalar invariance for the variable sex and social media use. Therefore, the aim of this study was to adapt and validate the MDTech-Q with a sample of Chilean university students.

## 2. Materials and Methods

### 2.1. Participants

Participants were selected using non-probability sampling. The sample was made up of 527 Chilean university students, including men (43.14%) and women (56.86%) enrolled in 12 Chilean universities. In relation to other sociodemographic characteristics of the participants, they are emerging adults [72] between 18 and 28 years (Mean = 22.09 years; SD = 3.59), whose socio-economic level is largely at low levels (47.4%) and medium levels (34.4%), while residence is mainly in urban areas (84.1%). Finally, 23.6% indicate belonging to an indigenous people group.

### 2.2. Instruments

First, a sociodemographic questionnaire made by the researchers was applied, the purpose of which was to collect demographic, age, educational, geographic, and other relevant data on the university students who participated in the study.

Second, the participants -Chilean university students- answered the Moral Disengagement through Technologies Questionnaire (MDTech-Q). This instrument assesses the cognitive mechanism of the MD that occurs in interactions through technology [13] based on the theoretical proposal and operationalization of the construct carried out by Bandura et al. [16]. This scale was adapted so that it addressed MD but in the online context and applied to a population of Spanish adolescents from Andalusia and is a Likert-type self-report scale composed of 16 items answered on a five-point scale (0 = Strongly disagree; 4 = Totally agree). It is made up of four correlated factors: Moral Justification through Technology (4 items, e.g., “Insulting or making fun of someone by cell phone or Internet to fight for something important is fine”), Diffusion of Responsibility through Technology (4 items, e.g., “In a WhatsApp group, Facebook, or other social network, people are not to blame for making fun of somebody if everybody is doing it”), Distortion of Consequences through Technology (4 items, e.g., “Actually, nothing serious happens to people when everybody makes fun of them on the Internet or cell phone”), Attribution of Blame through Technology (4 items, e.g., “If somebody takes a stupid photo or video of themself, it is their own fault if people share it by Internet or cell phone”). In addition, satisfactory indicators for reliability are reported (Moral Justification: α = 0.91; Diffusion of Responsibility; α = 0.91; Distortion of Consequences: α = 0.91; Attribution of Blame: α = 0.86), and validity (SB-χ^2^ (103) = 242.14 *p* < 0.001; RMSEA = 0.04 (90%IC = 0.03–0.04); CFI = 0.99; NFI = 0.99; NNFI = 0.99).

### 2.3. Procedure

The process of adapting the scale was guided by the criteria of the International Test Commission and other relevant authors in this area [73,74,75,76]. It is important to mention that the original scale is in Spanish, which is why the translation was not necessary, being reviewed by a panel of experts (methodologists, theoreticians, university professors and students) to take care that the items are adequately understood by Chilean university students. In this context, considering that in Chile the word “mobile” is not used to refer to personal telephones, this term was changed in the items that make up the scale deciding on the term “cell phone”. It was concluded that other changes to the questions were unnecessary, and the group interviews with university students were conducted to receive feedback on the understanding of the instructions and the content of the questionnaire prior to the application of the instruments.

Application of the scale was done online; therefore, the students received an e-mail with a link to the survey on the digital platform [77]. First, they accessed the informed consent to safeguard the ethical principles of this study and then they answered the survey.

### 2.4. Data Analysis

Descriptive measures of central tendency, dispersion, and shape of the distribution of each of the items on the scale were estimated. To evaluate the factorial structure of the MDTech-Q and to contrast its stability, two confirmatory factor analyses (CFA) were performed with the MPLUS v.7.1 software (Muthén & Muthén, Los Angeles, CA, USA) [78]. The estimation method used was the maximum likelihood estimation with robust standard errors (MLR). For the evaluation of CFA models, the following goodness-of-fit indices were used: SB-χ^2^, comparative fit index (CFI), Tucker-Lewis Index (TLI), root mean square error of approximation (RMSEA), and standardized root mean square residual (SRMR). For the CFI and TLI, an acceptable fit of the model was considered with values equal to or greater than 0.96, for the RMSEA values equal to or lower than 0.07 were considered a reasonable fit, whereas for the SRMR it was established at values less than or equal to 0.08 [79]. Then, a factorial invariance analysis was performed, which considered the following models [80]: M0 configural (equal number of factors), M1 metric (equal factor loadings), and M2 scalar (equality of intercepts). For the reliability estimation, the McDonald’s ω and Cronbach’s α coefficients were used [81,82].

## 3. Results

### 3.1. Descriptive Analysis

Table 1 provides the descriptive analysis of the mean of the items. These results show that the greatest value is in item 2, “If you are defending your friends, it is all right to annoy or upset somebody by cell phone or Internet” which corresponds to 1.91 (SD = 1.05). By contrast, the lowest mean value appears in item 11 “Insults by Internet or by cell phone do not hurt anybody”, which corresponds to 1.19 (SD = 0.49).

### 3.2. Factorial Structure

**Figure 1:**
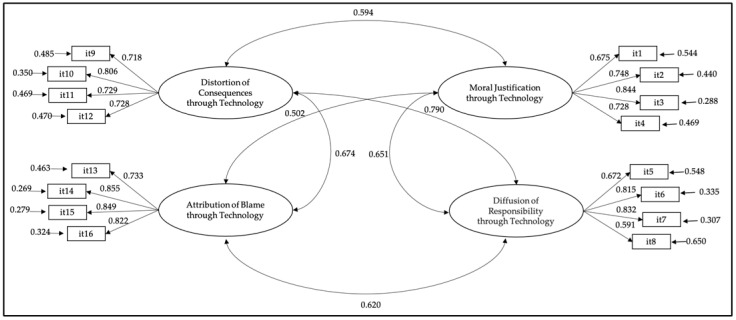
Four-factor correlated model. All the parameters were statistically significant (*p* < 0.001).

Two CFA models were estimated to evaluate the factorial structure of the MDTech-Q that contained the 16 items. The first evaluated model is unidimensional; it is relevant to indicate that this model showed an unsatisfactory fit [SB-χ^2^ (104) = 686.771 *p* < 0.001; CFI = 0.707; TLI = 0.662; RMSEA = 0.103 (90%CI = 0.096–0.110); SRMR = 0.087]. For the second, a four-factor correlated model was estimated; on this occasion, the results revealed an acceptable fit [SB-χ^2^ (103) = 153.588 *p* < 0.001; CFI = 0.972; TLI = 0.966; RMSEA = 0.33 (90%CI = 0.022–0.042); SRMR = 0.034)]. This reflects that the model presented next, with four correlated factors, fits well to the data and confirms the structure obtained by the CFA (Figure 1).

### 3.3. Factorial Invariance

Then, a factorial analysis of invariance (Table 2) was performed according to sex (0 = woman; 1 = man) and social media use (0 = 4 or more hours; 1 = 5 or more hours). The first contrasted model was M0 (Configuration invariance). Its result shows that the goodness-of-fit indices are satisfactory for both variables, making it possible to conclude that the factorial structure of the MDTech-Q presents factorial structure equivalence according to sex and social media use. Then, the M1 model (Metric invariance) was contrasted, which imposes restrictions on the factorial loads. The goodness-of-fit indices are satisfactory and the likelihood values show that there are no statistically significant differences between the M1 and M0 models. Therefore, the factorial loads are equivalent according to sex and social media use. Finally, the third M2 model was contrasted (Scalar invariance), which imposes restrictions on the intercepts, reflecting that there are no differences between M2 and M1, leading to the conclusion that the intercepts are equivalent according to sex and social media use.

Once the factorial equivalence between the groups had been determined, the mean differences were analyzed according to sex and social media use. As shown in Table 3, statistically significant differences were observed for all the factors of the MDTech-Q. According to sex, the highest means were obtained by the men in all the factors on the scale, having obtained a higher Cohen’s d (0.725) for the factor Attribution of Blame through Technology. No statistically significant differences were noted in the social media use.

### 3.4. Evidence of Reliability

In terms of the reliability of the scale (Table 4), the results of the four-factor correlated model show an acceptable reliability for each of the factors. The factor “Attribution of Blame through Technology” stands out for presenting the highest reliability values on the scale (ω = 0.889; α = 888; greatest lower bound (GLB) = 0.895).

## 4. Discussion

Society has progressively transformed over time, showing evidence of a significant increase in technological devices and Internet use, considering in particular the mass use of cell phones that allow people to be connected at any time and place [83]. Currently, people interact on different digital platforms, where violence, aggression, hate speech, cyberbullying, and other social phenomena that negatively affect people are observed [20,24,25,26,28,59], and these are important for Chilean university students since 78.3% use the social network daily [71]. MD through technologies is relevant because this construct is associated with negative Internet behaviors. In this sense, MD is contextualized, considering the characteristics of digital platforms that can favor the use of mechanisms that disconnect the behavior from the person’s moral values [13,43]. Therefore, this study sought to adapt and validate the Moral Disengagement through Technologies Questionnaire (MDTech-Q) with a sample of Chilean university students.

Regarding the first hypothesis, the results of this study showed that the MDTech-Q scores presented a factorial structure of four correlated dimensions, ruling out the unidimensional structure that could have been presented according to previous studies [16,61,63], in addition to evidence of good psychometric properties, specifically adequate indicators of validity and fit, as well as adequate reliability in Chilean university students (Moral Justification through Technology: ω = 0.839, α = 0.838; Diffusion of Responsibility through Technology: ω = 0.820, α = 0.815; Distortion of Consequences through Technology: ω = 0.834; α = 0.833; Attribution of Blame through Technology: ω = 0.889, α = 0.888). This scale is based on what was proposed by Bandura et al. [16]: The first mechanism of MD called moral justification addresses the personal and social resignification of a negative behavior, making it acceptable. The second mechanism called diffusion of responsibility suggests that people do not take responsibility for the negative action since this is shared or transferred to other people. The third mechanism called distortion of consequences is observed when the impact that negative behaviors can have on other people is minimized or ignored. The fourth mechanism called attribution of blame refers to the responsibility for the immoral behavior being shifted to the victim. The results are consistent with what was reported in the study that proposed this scale [13] since it demonstrates a factorial structure of four correlated dimensions, in addition to appropriate validity indicators and good reliability (Justification through Technology: α = 0.91; Diffusion of Responsibility through Technology: α = 0.91; Distortion of Consequences through Technology: α = 0.91; Attribution of Blame through Technology: α = 0.86).

With respect to the second hypothesis, a factorial analysis of invariance was performed, finding that the factorial structure of the MDTech-Q remains invariant up to the level of scalar invariance for the variable sex. This is relevant because differences are noted between men and women in the traditional MD and in the MD that emerges in online relationships, even though there are no conclusive results as other studies have found no differences [29,40,41,42,43,44,45,46,47,65]. In this study, differences were observed in the average scores obtained between men and women. The results show greater MD through technologies in the men, agreeing with studies that have assessed traditional MD [1,84,85]. This could be related to sociocultural aspects linked to aggression that would favor MD, such as women being socialized to be gentler than men, who are encouraged to be dominant and competitive. Therefore, aggression is more accepted in men, in addition to externalizing behaviors being observed more generally in men and prosocial behaviors more generally in women [17,41,42,86]. However, it is important to note that the study by Marín-López et al. [65] using this scale did not report any differences between men and women. On the other hand, in this study no statistically significant differences were found between the latent means for the variable social media use among the dimensions of the MD through Technologies construct.

Therefore, the MDTech-Q contributes by helping to understand interactions on the Internet, offering a scale that evaluates MD in a specific context such as the virtual environment with its own characteristics that differ from in-person spaces [13]. In addition, according to Paciello et al. [43], online MD might manifest cross-sectionally among people, whereas the traditional MD is associated with those who have behavioral problems. In this way, the MDTech-Q [13] will make it possible to collect more evidence using a scale adapted to interactions among people produced via information and communication technologies.

In the same vein, the relevance for the Latin American context of this instrument, its adaptation and validation with the Chilean university student population is worth noting, since in Latin America the contributions of MD in virtual environments use a scale relevant to its evaluation are limited, bearing in mind that the original study takes place with a sample of Spanish adolescents, therefore, in a different culture [13,65]. This refers to the social, economic, educational, political, and cultural conditions of Latin America [70], which are expressed in the interactions and information produced and disseminated on digital platforms on the Internet [66,67,87] reflecting the existing digital divide in this region [68,69].

In addition, this instrument has 16 items, and is considered brief, while it assesses the different MD strategies proposed by Bandura [16,18] related to the possibility of transforming the meaning of negative action by attributing little clarity regarding the responsibility of the harmful behavior, by minimizing and/or ignoring the consequences, and finally, by making the victim responsible.

The limitations of this study include the selection of the participants being carried out by non-probability sampling; however, university students from different geographic areas from central and southern Chile took part. On the other hand, it is important to point out that the applied scales are self-reporting and other instruments and interviews were not applied and no work was done with informants to contrast the information. In terms of future lines of research, the relations between MD through technologies and other constructs could be studied, such as cyberhate, trolling and so forth, due to the frequency with which negative and aggressive comments can be viewed on social networks. Along the same lines, it would be interesting to analyze its association with the construction of social identity by means of social networks [71], in light of the update of constructs in psychology that incorporate transformations in social interactions due to advances in technology and people’s adoption of them. Finally, it is relevant that this instrument can be applied in other countries and cultures to demonstrate if it maintains its factorial structure and to have the opportunity to compare the results, in addition to the possibility of an estimation of the test-retest reliability.

## 5. Conclusions

The adaptation and validation of the Moral Disengagement through Technologies Questionnaire (MDTech-Q) in a sample of Chilean university students provided adequate goodness-of-fit indices for the factorial structure and adequate reliability indices. Therefore, it is considered a contribution to evaluate Moral Disengagement, which addresses the cognitive processes used to disengage moral values, making it possible to justify inappropriate behaviors. Specifically, this instrument focuses on the interactions that occur in virtual contexts. This instrument highlights the relevance of technology and digital platforms on the Internet for most people.

## Figures and Tables

**Table 1:** Descriptive statistics for the items.

	Mean	Standard Deviation	Skewness	Kurtosis
1. Insulting or making fun of somebody by cell phone or Internet to fight for something important is fine.	1.61	0.83	1.26	0.92
2. If you are defending your friends, it is all right to annoy or upset somebody by cell phone or Internet.	1.91	1.05	1.01	0.10
3. If it is to get something good for your group, making fun of somebody by cell phone or Internet is justified.	1.46	0.68	1.58	2.69
4. If the honor of your group is at stake, it is justified to make insults by cell phone or Internet to defend it.	1.63	0.89	1.41	1.42
5. If everyone is doing it, the person is not to blame for passing on confidential information, harmful photos or videos by cell phone or Internet.	1.36	0.67	1.89	3.29
6. If nobody has banned it, people are not to blame for making fun of somebody by cell phone or Internet.	1.38	0.71	2.31	6.46
7. In a WhatsApp group, Facebook (or similar), people are not to blame for making fun of somebody if everybody is doing it.	1.31	0.62	2.40	6.96
8. It is not fair to blame a person for causing damage by cell phone or Internet if a lot of people are doing the same thing.	1.42	0.75	2.06	4.44
9. To make fun of people by Internet or cell phone does not really do any harm and is all right.	1.24	0.58	2.99	1.74
10. Actually, nothing serious happens to people when everybody makes fun of them on the Internet or cell phone.	1.22	0.55	3.13	12.41
11. Insults by Internet or cell phone do not anybody harm.	1.19	0.49	3.02	11.42
12. People who are insulted or ridiculed by Internet or cell phone easily forget it.	1.31	0.60	2.23	5.43
13. If somebody takes a stupid photo or video of themself, it is their own fault if people share it by Internet or cell phone.	1.64	0.99	1.59	1.79
14. People who are laughed at by cell phone or Internet are usually to blame for it.	1.56	0.83	1.49	1.91
15. People who are insulted by cell phone or Internet have brought it on themselves.	1.54	0.78	1.46	1.89
16. People who are not careful with their personal data (photos, passwords, secrets) are to blame if somebody steals it and it gets shared by cell phone or Internet.	1.61	0.90	1.55	1.87

**Table 2:** Factorial invariance.

Variable	Model	SB − χ^2^ (df)	CFI	TLI	RMSEA	Comp.	ΔSB − χ^2^	Δgl	*p* (ΔSB − χ^2^)
Sex	1 M0	279.732 (196)	0.958	0.948	0.040				
	2 M1	287.340 (208)	0.960	0.954	0.038	2 vs. 1	9.223	12	0.684
	3 M2	306.161 (220)	0.956	0.952	0.039	3 vs. 2	19.665	12	0.0737
Social media use	1 M0	300.568 (196)	0.954	0.943	0.045				
	2 M1	305.138 (208)	0.957	0.950	0.042	2 vs. 1	6.353	12	0.8973
	3 M2	317.230 (220)	0.957	0.953	0.041	3 vs. 2	9.219	12	0.6841

comp. means model comparison; (df) means degrees of freedom.

**Table 3:** Independent samples *t*-test.

	Sex		Social Media Use	
Factors	Student’s *t*-Test (df)	Cohen’s d	Student’s *t*-Test (df)	Cohen’s d
Moral Justification through Technology	5.292 ** (527)	0.473	−1.123 (527)	−0.098
Diffusion of Responsibility through Technology	3.787 ** (527)	0.338	0.280 (527)	0.024
Distortion of Consequences through Technology	4.603 ** (527)	0.411	−0.751 (527)	−0.065
Attribution of Blame through Technology	8.113 ** (527)	0.725	1.082 (527)	0.094

**Note**. ** *p* < 0.001.

**Table 4:** Evidence of reliability according to factors.

	McDonald’s ω	Cronbach’s α	Greatest Lower Bound (GLB)
Moral Justification through Technology	0.839	0.838	0.883
Diffusion of Responsibility through Technology	0.820	0.815	0.850
Distortion of Consequences through Technology	0.834	0.833	0.848
Attribution of Blame through Technology	0.889	0.888	0.895

## Data Availability

The data presented in this study are available on request from the corresponding author.
